# Optimizing nitrogen management in grain rotations: balancing retention and photosynthesis

**DOI:** 10.3389/fpls.2026.1761467

**Published:** 2026-04-02

**Authors:** Dan Liu, Yudong Zheng, Xin Hui, Xuetong Liu, Caiyun Cao, Kejiang Li, Hongkai Dang, Chunlian Zheng

**Affiliations:** 1 Key Laboratory of Crop Drought Resistance Research of Hebei Province, Institute of Dryland Farming, Hebei Academy of Agriculture and Forestry Sciences, Hengshui, China; 2 Key Laboratory of Crop Physio-ecology and Tillage Science in the Northwestern Loess Plateau, Ministry of Agriculture/College of Agronomy, Northwest A&F University, Xianyang, Shaanxi, China; 3 Water Conservancy and Architectural Engineering, Northwest A&F University, Xianyang, Shaanxi, China

**Keywords:** garlic-maize rotation system, nitrogen allocation dynamics, nitrogen utilization, nitrogen distribution, photosynthetic coupling

## Abstract

Diversified cropping systems exhibited enhanced resource utilization efficiency, yet the nitrogen (N) allocation mechanisms and utilization patterns in economic-grain rotation systems remain poorly understood. This study investigated the cross-seasonal N allocation dynamics and photosynthetic responses to N reduction in a garlic–maize rotation system through a three-season field experiment with graded N treatments (garlic, 300 and 240 kg N ha^−1^; maize, 220, 175, and 130 kg N ha^−1^). N reduction increased soil water-filled pore space (WFPS) by 5.2%–8.7% during maize seasons but decreased it in garlic seasons. It also reduced topsoil (0–20 cm) NO_3_^−^–N accumulation by >15% compared to deeper layers. Leaf physiological parameters—including leaf area index (LAI), SPAD values, and net photosynthetic rates—declined by 18%–32% under N reduction, with garlic demonstrating higher sensitivity. Residual N from preceding garlic crops stabilized maize LAI (± 6.5%), indicating compensatory inter-season adjustments. Critical thresholds were identified: maize achieved optimized grain nitrogen partitioning (65% to 72%) and a 22% improvement in nitrogen use efficiency (NUE) with a reduction of 45 kg N ha^−1^ without a significant yield penalty (less than 5%). Conversely, garlic experienced a 23% increase in stem nitrogen translocation when nitrogen was reduced by 60 kg N ha^−1^, which resulted in a 34% decrease in bulb allocation. Annual N reduction (8.65%–28.85%) enhanced maize agronomic efficiency (+40%) but reduced garlic yields (4.2%–27.5%) and partial factor productivity (−18%). These results reveal contrasting, crop-specific N allocation strategies and support the development of demand-driven N management to balance productivity and economic outcomes in multi-cropping systems.

## 1 Introduction

Diversified cropping systems, occupying 12% of global arable land, deliver over 873.95 billion kg of food annually while critically supporting global food security (Waha et al., 2020; Londoño Parra, 2023; Taras et al., 2021). Optimizing nutrient management in these systems requires aligning fertilization strategies with crop-specific demands to reduce input without compromising productivity (Araya et al., 2021; Kaur et al., 2024). As the most prevalent agrochemical, nitrogen (N) fertilizers contribute ~50% to global yield increases (Munasinghe–Arachchige and Nirmalkanda, 2020). However, China’s accelerated N consumption contrasts with stagnant agronomic efficiency, underscoring the urgency for strategic reduction (World Bank, 2016; Luo et al., 2024). While moderated N applications conserve yield efficiency, achieving seasonally balanced N allocations remains challenging in economic-grain rotations—a system enhancing productivity and ecosystem resilience (Daly and Hernandez–Ramirez, 2020; You et al., 2023). Therefore, further exploration is needed to balance the N requirements of each crop growth season to achieve the optimal production in multiple cropping systems (Garbelini et al., 2022; Liu et al., 2024). Excessive N inputs during cash crop phases create dual challenges: inefficient utilization and environmental leaching, and incomplete accounting of residual N impacts (Zhao et al., 2020; Rose et al., 2023).

Physiologically, optimal N levels enhance photosynthetic capacity through increased leaf area index and chloroplast density (Jafarikouhini et al., 2020), thereby improving light interception and assimilating partitioning to harvest organs (Gao et al., 2023). Both N deficiency and excess disrupt this equilibrium—the former limits growth through impaired photomorphogenesis (Kang et al., 2023), while the latter extends vegetative phases and reduces harvest index (Wang et al., 2020). Soil moisture-mediated NO_3_^−^–N dynamics further complicate N management, with water-filled pore space (WFPS) exhibiting contradictory effects: enhancing nitrification rates (Liang et al., 2022) versus promoting plant uptake dilution (Krevh et al., 2023). N application changes the concentration of NO_3_^−^–N, affecting crop uptake of soil nitrogen (Li et al., 2023). During this process, soil moisture participates in and affects the formation and distribution of NO_3_^−^–N (Shi et al., 2022). At present, the influence of soil moisture on NO_3_^−^–N is controversial. Appropriate soil moisture will enhance nitrification and increase NO_3_^—^N. However, it may also enhance the crop’s ability to absorb and dilute nitrogen, decreasing NO_3_^−^–N (Liang et al., 2022; Krevh et al., 2023). Adjusting the N application rate will affect the concentration of nitrifying substrates, further increasing the complexity of changes in NO_3_^−^–N (Li et al., 2023). Therefore, clarifying the relationship between moisture and NO_3_^−^–N, as well as their impact on crop photosynthesis during the growing season, can provide scientific evidence for soil environmental changes and crop physiological responses.

As a cash crop, garlic has the function of alleviating continuous cropping obstacles or balancing soil nutrients in the multiple cropping system (Alam et al., 2019). In some regions, the intercropping of garlic and grain crops is a high-yield and diversified production system, but excessive nitrogen application also affects the stability of the agricultural system’s ecological environment (Jaggi and Raina, 2008; Tang et al., 2012; Wang et al., 2022). Current evidence regarding economic-grain rotation systems remains fragmented. Most studies have focused only on nitrogen reduction within a single season or solely on yield and nitrogen use efficiency (NUE). The cross-seasonal carryover of residual nitrogen and its impact on crop-specific nitrogen allocation priorities are rarely quantified. Additionally, the interaction between soil water and nitrate, which regulates NO_3_^−^–N availability, is often treated as a confounding background factor rather than a direct driver of photosynthetic performance. This leaves the influence of changes in WFPS and NO_3_^−^–N on photosynthesis, dry matter/nitrogen partitioning, and yield trade-offs across successive cash and grain crop seasons unclear. Therefore, we designed a maize–garlic multiple cropping experiment in the Guanzhong region, reducing nitrogen levels by 60 kg ha^−1^ (G_240_) during the garlic growing season, and 45 kg ha^−1^ (M_175_) and 90 kg ha^−1^ (M_130_) during the maize growing season, based on the local conventional nitrogen application rate G_300_M_220_ (300 kg ha^−1^ for garlic and 220 kg ha^−1^ for maize). By measuring the soil WFPS and NO_3_^−^–N in the 0–40-cm soil during the crop growth period, the photosynthetic capacity of plants during key growth periods, the distribution characteristics of dry matter and nitrogen absorption, crop yield, and calculating nitrogen utilization efficiency, a structural equation model elucidated the N reduction impacts across soil–crop physiological interfaces. This multidisciplinary approach aims to 1) quantify soil N–water coupling under gradient N regimes, 2) decipher WFPS/NO_3_^−^–N effects on photosynthetic thresholds, and 3) establish system-level N allocation strategies balancing economic-grain priorities. We hope to establish an optimized nitrogen (N) management framework for economic-grain rotation systems by modulating annual N inputs based on real-time soil nitrate dynamics and crop-specific photosynthetic responsiveness and nitrogen assimilation efficiency.

## 2 Materials and methods

### 2.1 Study site

**Figure 1:**
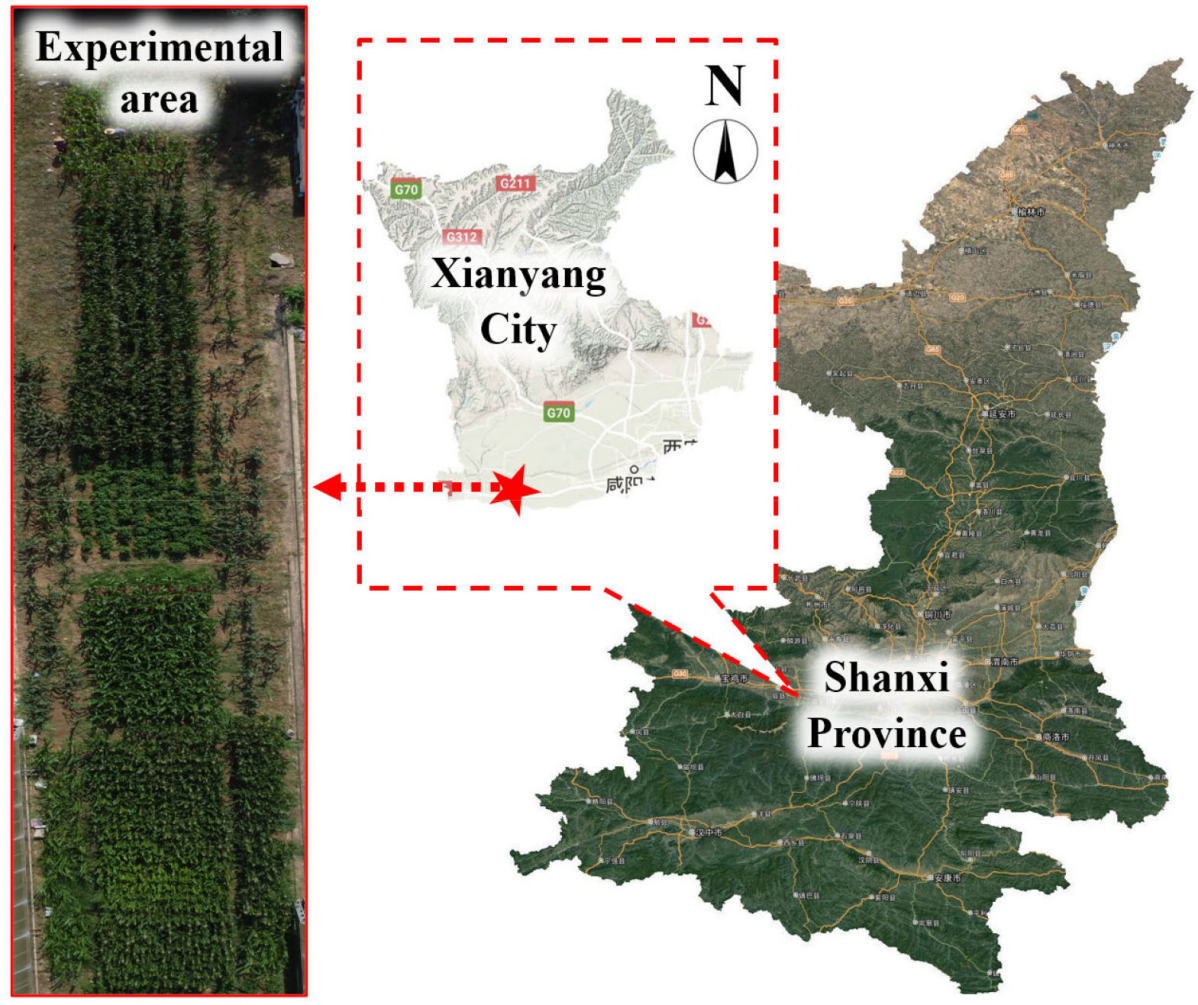
Geographical location of the experimental site.

**Figure 2:**
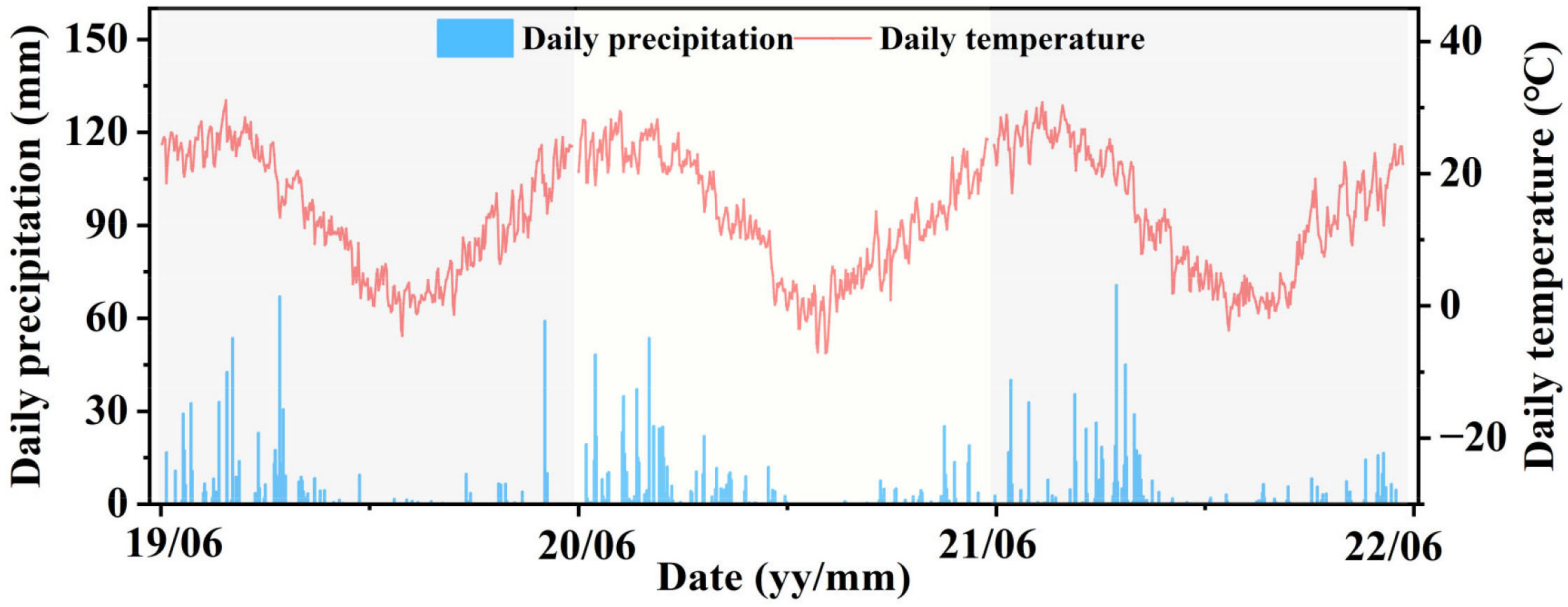
Precipitation and daily average temperature during the experiment.

The experiment was conducted in the field from June 2019 to May 2022 at the Institute of Water-Saving Agriculture in Arid Areas of China, Northwest A&F University, China (34°21′N, 108°10′E, 524.7 m asl). The site is located in the hinterland of the Guanzhong Plain, which is a semi-humid and drought-prone area, and the planting system involves two crops each year (**Figure 1**). The growing season for maize is from June to September, and the growing season for garlic is from October to May. **Figure 2** shows the daily mean temperature and precipitation during the crop growing stages. The 0–20 cm-layer of the top soil contained 16.56 g kg^−1^ organic matter, 45.37 mg kg^−1^ alkali-hydrolyzed N, 13.29 mg kg^−1^ available phosphorus, and 109.62 mg kg^−1^ available potassium. The soil bulk density was 1.32 g cm^−3^, and the pH was 7.20 (Liu et al., 2023).

### 2.2 Experimental design and field planting

The garlic–maize multiple cropping system experiment was designed as a single-factor completely randomized block trial. Two levels comprising conventional N application at 300 kg ha^−1^ (G_300_) and 20% less N application at 240 kg ha^−1^ (G_240_) were tested in the garlic growing season. Three levels comprising conventional N application at 220 kg ha^−1^ (M_220_), 20% less N application at 175 kg ha^−1^ (M_175_), and 40% less N application at 130 kg ha^−1^ (M_130_) were tested in the maize season. Thus, six treatments were tested, comprising G_300_M_220_, G_300_M_175_, G_300_M_130_, G_240_M_220_, G_240_M_175_, and G_240_M_130_, with three replicate plots for each treatment and a total of 18 plots. The area of each plot was 24 m^2^ (4 m × 6 m). Phosphorus fertilizer (P_2_O_5_: 150 kg ha^−1^) and potassium fertilizer (K_2_O: 150 kg ha^−1^) were applied to the soil in both seasons as basal fertilizer during the sowing period. In the two maize growing seasons, N fertilizer was applied to the soil in a 5-cm-deep furrow with topdressing on July 29, 2019, June 29, 2020, and June 23, 2021. In the first garlic growing season, N fertilizer was applied to the soil once in a 5-cm-deep furrow on May 8, 2020. In the second and third garlic growing seasons, 50% of the N fertilizer was applied to the soil as a base fertilizer by rotary tillage (15-cm depth) on September 24, 2020, and October 20, 2021, and the other 50% was applied to the soil in a 5-cm-deep trench as topdressing on March 20, 2021, and March 25, 2022. The specific fertilization methods are shown in **Supplementary Table 1**.

The garlic variety Cangshan was sown on September 23, 2019, September 24, 2020, and September 25, 2021, with a planting density of 400,000 ha^−1^ plant (row spacing = 25 cm, plant spacing = 10 cm), and harvested on May 22 in the following year. The maize variety Zhengdan 958 was sown on May 22, 2019, May 24, 2020, and May 27, 2021, with a planting density of 66,667 plants ha^−1^ (row spacing = 60 cm, plant spacing = 25 cm) and harvested on September 21. Weeding, pest control, and irrigation were performed during the trial according to the local agricultural management practices.

### 2.3 Soil water-filled pore space and NO_3_^−^–N concentration

Soil samples of 0–40 cm were taken from each treatment during critical stages of crop growth and before fertilization. A portion of the soil samples was dried to measure soil mass moisture content, and WFPS was calculated based on soil bulk density (Thilakrathna and Hernandez−Ramirez, 2021). The other part of fresh soil samples passed through a 2-mm sieve, and the concentration of NO_3_^−^–N was determined using a flow analyzer (AutoAnalyzer 3-AA3, SEAL Analytical, Norderstedt, Germany).

### 2.4 Leaf area index, SPAD, and photosynthetic rate

The total leaf area at the flower bud differentiation stage (FBS) and bulb enlargement stage (BES) of garlic, the 12-leaf stage (V12), and silking stage (R1) of maize was measured and calculated according to the method of Aldesuquy et al. (2014), and the leaf area index (LAI) was calculated using Equation 1 based on the unit land area:

(1) LAI=∑(Leaf length×Leaf width)×0.75×planting density

where the leaf length and width were measured for all fully expanded green leaves (m) of each plant, 0.75 was the calculation coefficient of leaf area, and the planting density was calculated according to the unit land area (plant m^−2^). While measuring the leaf area, the relative chlorophyll values of the base, middle, and tip of each leaf were measured using a portable SPAD-502 chlorophyll meter (Konica Minolta, Inc., Tokyo, Japan), and the average relative chlorophyll value of the plant leaves was calculated.

In the garlic growing season, the research method of Dong et al. (1993) was improved. The sunny weather was selected, and the GXH-305 infrared CO_2_ analyzer was used for measuring in the field at 9:00–11:00 a.m. The box for measuring the photosynthetic assimilation of the colony was composed of two parts: the box and the base. The height of the box was 1.2 m, the length was 0.8 m, the width was 0.7 m, the outer cover was a transparent polyester plate, and the transmittance was 95%. During the measurement, the lower base tank was filled with water to seal the box. The canopy apparent photosynthesis (CAP) was calculated using Equation 2:

(2) $$\text{CAPμ}{\text{mol CO}}_{2}{\text{ m}}^{-2}{\text{s}}^{-1}=\text{ΔC}\times \frac{\text{V}}{10}-6\times \frac{60}{△m}\times \frac{44}{22.4}\times 6.313\times \frac{273}{\left(273+T\right)}/\text{S}$$

where △C is the difference of CO_2_ concentration measured successively in the interval time (μL L^−1^), V is the assimilation chamber volume (L), △m is the measurement time (min), *T* is the average temperature in the assimilation chamber (°C), and S is the area of land occupied by the group (m^2^).

In the growing season of maize, the net photosynthetic rate (Pn) of maize functional leaves was measured using a portable photosynthesis instrument Li-6400 (Li-COR Inc., Lincoln, NE, USA) in sunny weather from 9:00 to 11:00 am.

### 2.5 Accumulation and distribution of dry matter and nitrogen in crops

Ten garlic plants were sampled in the FBS and BES for each treatment. The samples were dried in an oven at 105 °C for 30 min and then dried to constant weight at 75 °C to determine the dry matter mass. The above-ground maize plant parts were sampled in the V12 and R1, with three replicates for each treatment. The drying and weighing methods were the same as those used for garlic.

The dried plant samples were broken into powder according to different organs. After digestion reaction, the N content in the samples was determined using a Kjeltec 8400 automatic analyzer (FOSS Analytical AB, Höganäs, Sweden), and the N absorption and accumulation in different organs were calculated according to the dry matter quality.

### 2.6 Crop yield and nitrogen uptake

Garlic stems and bulbs were collected at the harvest, with three replicates for each treatment and two rows from each plot, and the fresh weights were recorded. Ears were harvested from maize plants in the maturity stage, with three replicates for each treatment. Two rows were harvested from each plot. After threshing, air drying, and weighing, the yield was calculated at a moisture content of 14%. The nitrogen use efficiency (NUE), partial factor productivity of nitrogen (PFPN), and agronomic efficiency of nitrogen (AEN) were calculated using Equations 3–5, respectively.

(3) Nitrogen use efficiency (NUE,%)=(U−U0)/F

where U is the total nitrogen content of the above-ground part of the crop after fertilization, U0 is the total nitrogen content of the above-ground part of the crop during the harvest period without fertilization (see **Supplementary Table 2**), and F represents the amount of fertilizer input.

(4) $$\text{Partial factor productivity from applied N }(\text{PFPN},{\text{kg kg}}^{-1}),\text{PFPN}=\text{Y}/\text{F}$$

where Y represents the crop yield of nitrogen treatment, and F represents the amount of fertilizer input.

(5) $$\text{Agronomic efficiency of applied N }(\text{AEN},{\text{kg kg}}^{-1}),\text{AEN}=\left(\text{Y}-\text{Y}0\right)/\text{F}$$

where Y represents the crop yield of nitrogen treatment, Y0 represents the crop yield without nitrogen treatment, and F represents the amount of fertilizer input.

### 2.7 Statistical analyses

Statistical analyses were performed using SPSS 25.0 (IBM Corp., Armonk, NY, USA). Duncan’s multiple comparison method in one-way ANOVA analysis was used to test the significance at a *p* < 0.05 level. According to the method described by You et al. (2020), the SmartPLS 4.0 (University of South Alabama, USA) software was used to conduct path analysis based on the water-filled pore space, NO_3_^−^–N contents, dry matter, leaf photosynthetic characteristics, yield, and nitrogen uptake due to fertilizer application.

## 3 Results

### 3.1 Soil water-filled pore space

**Figure 3:**
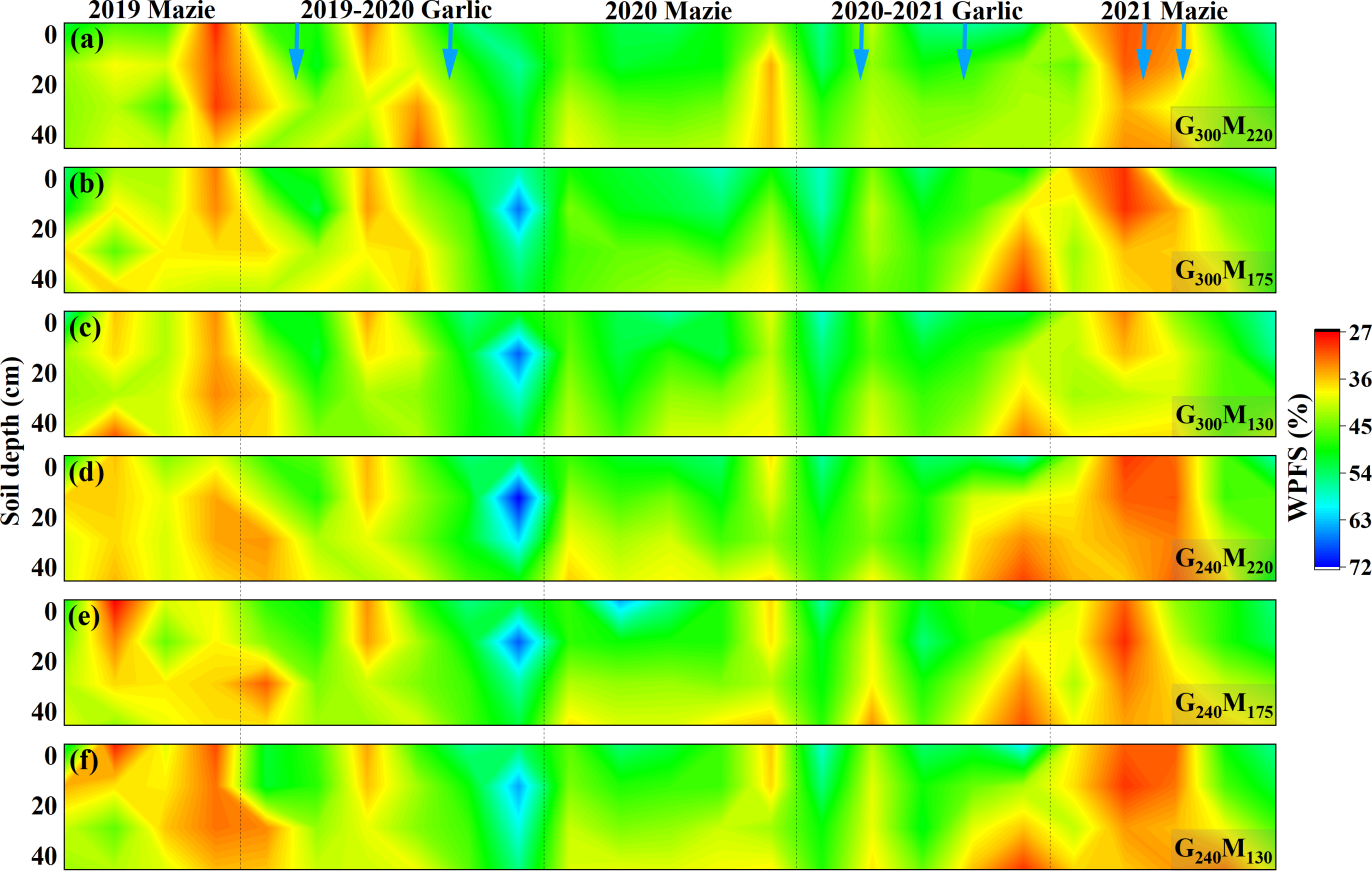
The spatial distribution of WFPS in 0−40 cm soil with different fertilization treatments changed with planting time. The blue arrow in the figure indicates supplementary irrigation, and the amount of irrigation each time is 100 mm.

The response of 0–40-cm soil WFPS to N application was different during the growth season of maize and garlic crops. Reducing the N application rate during the maize growing season increased soil WFPS (**Figures 3a–c**). Compared with G_300_M_220_, G_300_M_175_ and G_300_M_130_ increased soil WFPS by 1.40% and 2.60%, respectively, in the 0–40-cm soil layer during the planting period. Compared to G_240_M_220_, G_240_M_175_ had a 1.10% increase in WFPS. However, reducing the N application rate during the garlic growing season reduced soil WFPS (**Figures 3d–f**), and reducing 60 kg N ha^−1^ during the garlic growing season decreased WFPS by 1.75%–4.22%. In summary, reducing N application increased soil WFPS in the maize season but decreased it during the garlic season, indicating a contrasting water response between the two crops.

### 3.2 Soil NO_3_^−^–N

**Figure 4:**
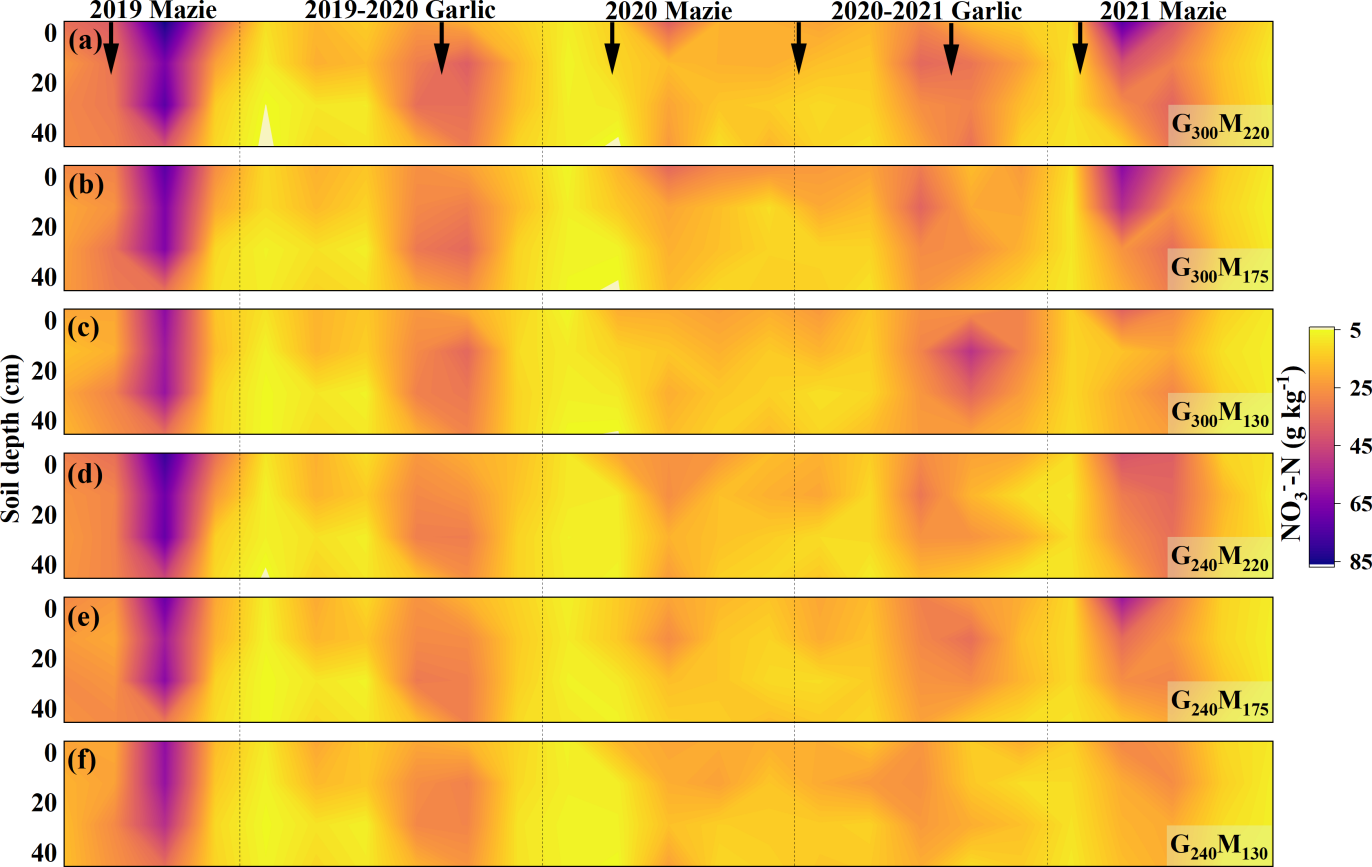
The spatial distribution of NO_3_^−^– concentration in 0−40 cm soil with different fertilization treatments changed with planting time. The red arrow in the figure indicates nitrogen application.

The soil NO_3_^−^–N significantly increased after N application and showed a decreasing trend with the N reduction in 0–40-cm soil (**Figure 4**). Compared to the garlic growing season, the concentration of NO_3_^−^–N in the maize growing season was higher. The effect of N application frequency and amount on NO_3_^−^–N was relatively small in the two growth seasons of garlic. Compared with G_300_M_220_, reducing N application rates by 8.65%–28.85% decreased NO_3_^−^–N by 5.49%–21.18%. The decrease in NO_3_^−^–N in the surface soil was greater than that in the lower soil, with a decrease of 4.19%–12.84% in the 0–20-cm soil layer and a decrease of 2.41%–8.66% in the 20–40-cm soil layer. Compared with the garlic growing season, reducing N fertilizer has a stronger inhibitory effect on NO_3_^−^–N during the maize growing season. Overall, N reduction consistently lowered soil NO_3_^−^–N content, with a more pronounced decrease in the maize season and in the surface soil layer.

### 3.3 Leaf photosynthetic characteristics

**Figure 5:**
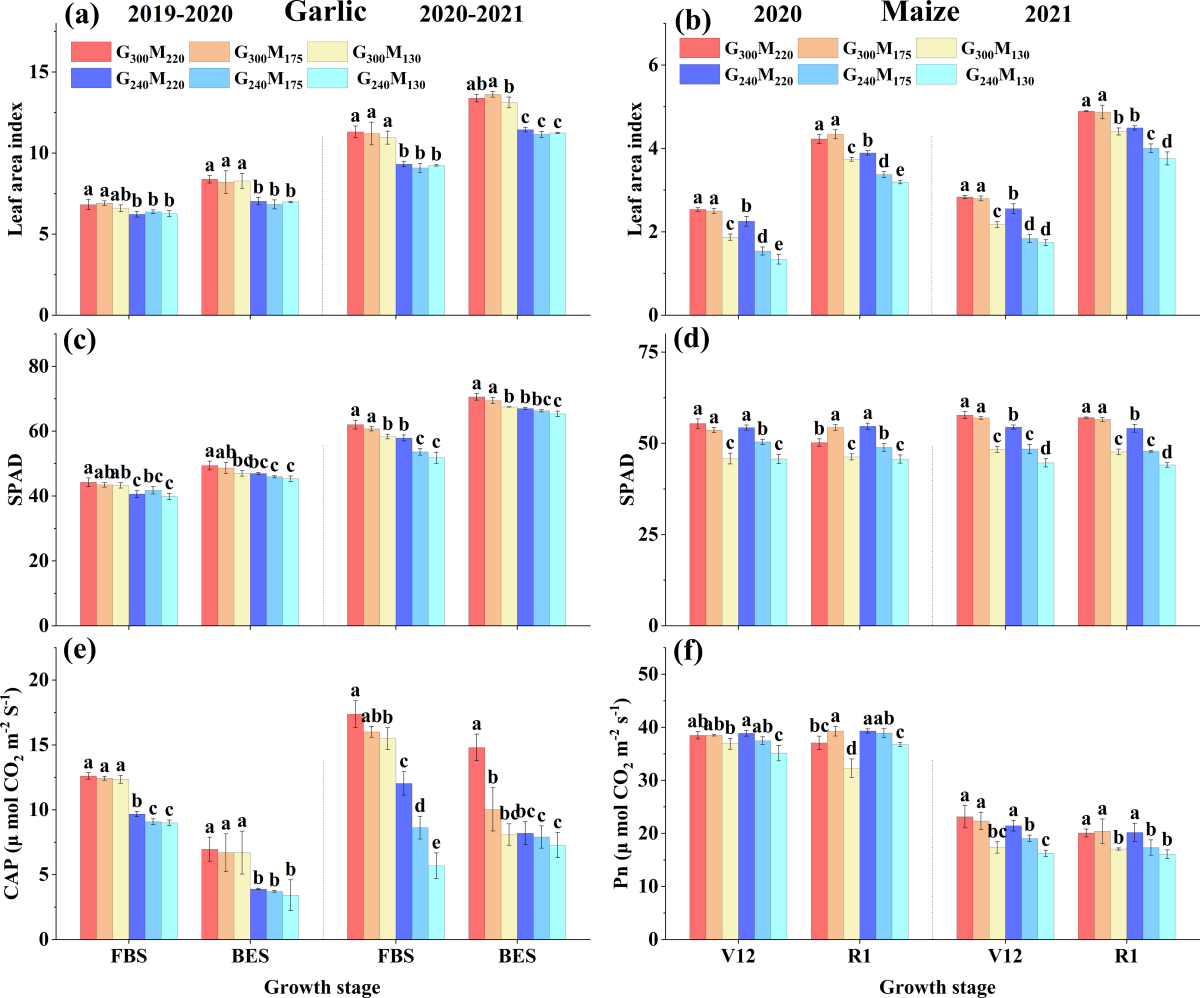
Leaf area index, SPAD and net photosynthetic rate of garlic and maize under different nitrogen treatments. CAP represents the canopy apparent photosynthetic rate of garlic; Pn represents the net photosynthetic rate of maize functional leaves. The absence of the same lowercase letters on the column indicated a significant difference between different treatments at the *P* < 0.05 level.

There were differences in the response of LAI and SPAD to N reduction during the growth period of the two crops. During the garlic growing season, reducing 60 kg N ha^−1^ significantly decreased the LAI by 12.29% and 15.94%, as well as the SPAD by 8.26% and 4.46% in the FBS and BES, respectively (**Figures 5a, c**). During the maize growing season, reducing 45 kg N ha^−1^ in the previous crop at 300 kg ha^−1^ had no significant impact on LAI and SPAD. However, at 240 kg ha^−1^ in the previous crop, it significantly decreased LAI by 29.88% and 12.10%, and SPAD by 9.15% and 11.13%, respectively, in the V12 and R1. Reducing 90 kg N ha^−1^ in the current season further significantly decreased the LAI and SPAD of maize (**Figures 5b, d**). Reducing 60 kg N ha^−1^ significantly decreased the CAP of garlic by 36.26% and 35.64% in the FBS and BES, respectively, and the CAP decreased with the N reduction in the previous maize growing season. Reducing 45 kg N ha^−1^ in the current season had little effect on the Pn of maize, while reducing 90 kg N ha^−1^ significantly decreased Pn by 15.76% and 13.69% in the V12 and R1, respectively. Taken together, N reduction negatively affected photosynthetic traits in both crops, although the sensitivity of LAI, SPAD, and gas exchange parameters varied with crop type and prior N management.

### 3.4 Accumulation and distribution of dry matter and nutrients

**Figure 6:**
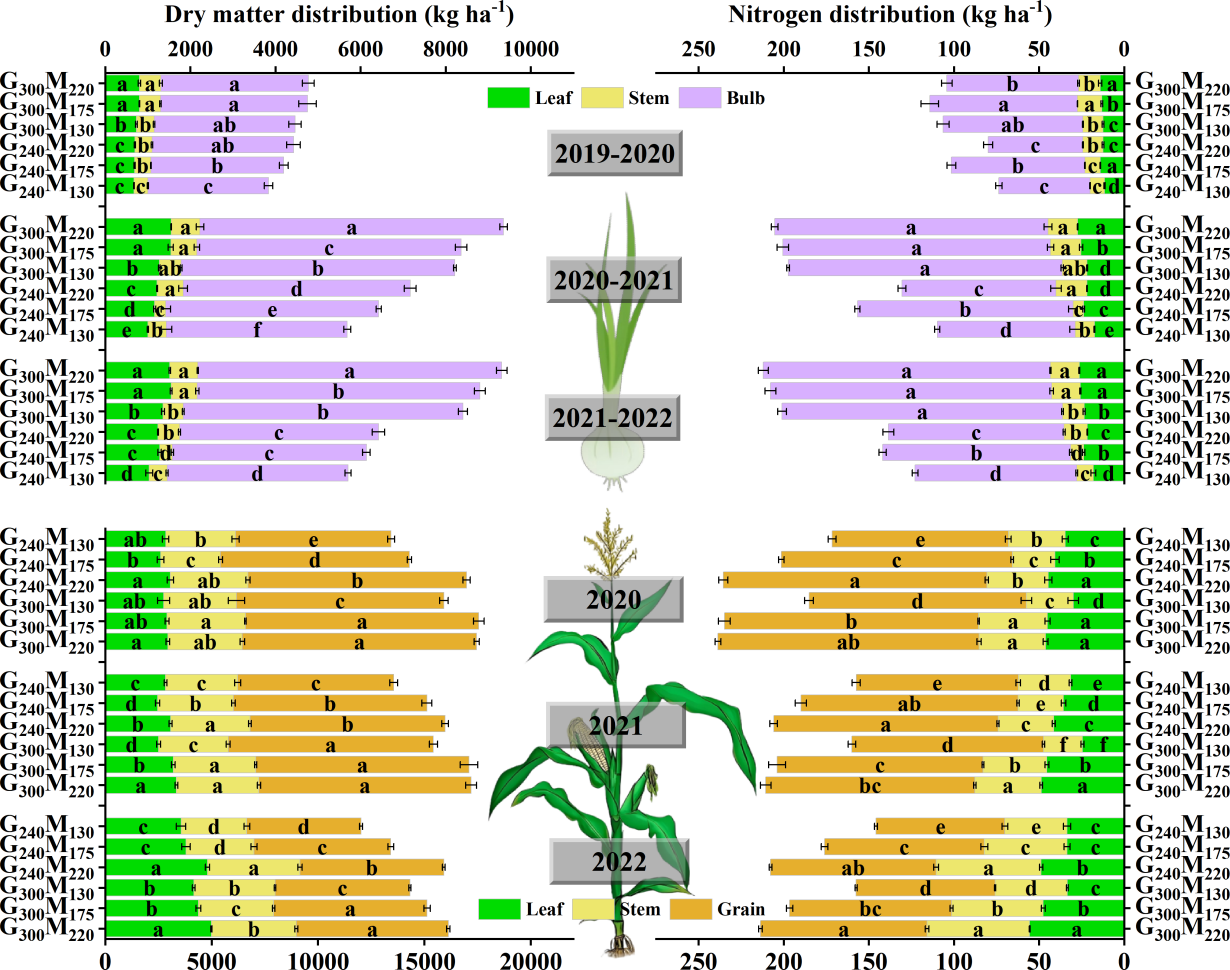
Absorption and distribution of dry matter and nitrogen in garlic and maize under different nitrogen treatments. The absence of the same lowercase letters on the column indicated a significant difference between different treatments at the *P* < 0.05 level.

Compared with G_300_M_220_, a reduction of 8.65%–28.85% annual N application decreased the dry matter accumulation of garlic by 5.53%–29.41%, with a decrease in the ratio of leaf dry matter and an increase in the ratio of bulb dry matter (**Figure 6a**). Under the N application rate of 300 kg ha^−1^ in the garlic season, reducing N in the previous maize growing season increased the N absorption rate of garlic bulbs. Decreasing the N absorption rate to 90 kg ha^−1^ in the maize growing season and 60 kg ha^−1^ in the garlic growing season (G_240_M_130_) significantly decreased the N absorption rate of garlic and garlic bulbs (**Figure 6b**). Compared with G_300_M_220_, except for G_300_M_175_, other N reduction treatments significantly decreased the dry matter accumulation of maize by 9.55%–22.11% (**Figure 6c**). The effect of reducing 45 kg N ha^−1^ in the maize season or 60 kg N ha^−1^ in the garlic season on the N uptake of maize was not significant (**Figure 6d**). When applying 300 kg N ha^−1^ in the garlic growth season, reducing the N application rate in the maize season increased the dry matter mass and N absorption rate of the grains. G_240_M_130_ significantly decreased the dry matter mass and nitrogen absorption rate of grains. In brief, reducing N application generally reduced dry matter accumulation and altered its partitioning, while N uptake was influenced by both current and previous season N rates.

### 3.5 Crop yield and nitrogen use efficiency

**Table 1** shows that, compared to G_300_M_220_, except for G_300_M_175_, a significant reduction of 11.54%–28.85% in nitrogen application significantly reduced maize yield by 10.84%–29.38%. Moreover, reducing 60 kg N ha^−1^ in the previous crop further decreased maize yield. Compared with G_300_M_220_, the annual N application rate reduced by 8.65%–28.85% and significantly decreased garlic yield by 4.23%–27.52%, and the decreasing trend of garlic yield increased with the reduction in N in the previous maize season. Reducing 45 kg N ha^−1^ in the maize season significantly increased the NUE by 18.28%, while reducing the N application rate by 11.54%–28.85% decreased the NUE by 3.93%–31.04%. Reducing the N application rate in the maize growth season increased the partial factor productivity of nitrogen (PFPN) and agronomic efficiency (AEN), but reducing the N application rate in the previous crop further decreased the PFPN and AEN of the maize. Drought caused garlic production to be significantly lower in the first year than in other years and decreased the differences in NUE, PFPN, and AEN between different N application treatments. As the N application rate decreased, the NUE, PFPN, and AEN of garlic showed a downward trend. In the second year, compared to G_300_M_220_, the reduced N application rate of 8.65%–28.85% decreased NUE by 3.06%–49.65%, PFPN by 4.89%–20.97%, and AEN by 8.47%–54.61%. Overall, N reduction tended to decrease yield in both crops, while its effect on NUE depended on the crop and the extent of N reduction, with maize showing some potential for improved NUE under moderate reduction.

**Table 1:** Crop yield and nitrogen use efficiency under different nitrogen application rates.

Crops	Treatments	Yield (kg ha^−1^)	NUE (%)	PFPN (kg kg^−1^)	AEN (kg kg^−1^)
2019 maize	G_300_M_220_	6,572.8 a	38.8 b	29.9 d	16.5 b
	G_300_M_175_	6,880.5 a	46.7 a	39.3 b	22.5 a
	G_300_M_130_	5,625.3 b	30.4 c	43.3 a	20.6 a
	G_240_M_220_	5,953.6 b	37.7 b	27.1 e	13.7 d
	G_240_M_175_	5,799.9 b	38.0 b	33.1 c	16.3 cd
	G_240_M_130_	4,936.9 c	26.4 d	38.0 b	15.3 cd
2019–2020 garlic	G_300_M_220_	6,682.0 a	22.5 b	22.6 c	13.1 b
	G_300_M_175_	6,449.6 b	25.8 a	21.9 d	12.4 c
	G_300_M_130_	6,297.6 bc	23.2 b	21.5 d	12.0 c
	G_240_M_220_	6,053.3 d	18.0 c	25.7 a	13.9 a
	G_240_M_175_	6,085.5 cd	27.0 a	25.8 a	14.0 a
	G_240_M_130_	5,584.9 e	15.4 d	23.8 b	11.9 c
2020 maize	G_300_M_220_	7,347.3 a	41.2 b	32.7 d	17.6 cd
	G_300_M_175_	7,246.8 a	47.6 a	41.4 b	22.6 b
	G_300_M_130_	6,684.5 b	30.3 c	50.5 a	25.4 a
	G_240_M_220_	6,889.1 b	39.1 b	30.8 d	15.7 d
	G_240_M_175_	6,572.7 b	39.7 b	38.6 c	19.8 c
	G_240_M_130_	5,296.0 c	28.4 c	41.7 a	16.6 d
2020–2021 garlic	G_300_M_220_	23,651.3 a	53.3 a	80.8 a	46.6 a
	G_300_M_175_	23,147.9 a	51.7 ab	76.8 a	42.7 a
	G_300_M_130_	21,502.9 b	50.7 b	70.3 b	36.1 b
	G_240_M_220_	19,001.0 c	35.5 d	77.4 a	34.8 b
	G_240_M_175_	17,262.5 d	46.4 c	70.7 b	28.0 c
	G_240_M_130_	15,293.6 e	26.9 e	63.8 c	21.2 d
2021 maize	G_300_M_220_	10,875.2 a	52.1 b	49.0 d	30.9 c
	G_300_M_175_	10,941.4 a	63.0 a	66.2 b	43.6 a
	G_300_M_130_	9,913.0 c	46.3 c	75.0 a	44.8 a
	G_240_M_220_	10,361.8 b	50.7 b	48.7 d	30.6 c
	G_240_M_175_	9,506.6 c	44.0 c	54.3 c	31.7 c
	G_240_M_130_	8,913.9 d	40.0 d	67.1 b	36.9 b
2021–2022 garlic	G_300_M_220_	24,165.0 a	54.8 a	80.5 a	43.0 a
	G_300_M_175_	22,451.2 b	53.5 a	74.8 b	37.3 b
	G_300_M_130_	19,606.7 c	51.4 b	65.4 c	27.8 c
	G_240_M_220_	19,462.7 c	36.9 d	81.1 a	34.2 b
	G_240_M_175_	18,323.2 d	39.5 c	76.3 b	29.5 c
	G_240_M_130_	16,721.1 e	31.3 e	69.7 c	22.8 d

Different lowercase letters in the table indicate that the difference between treatments is significant at *p* < 0.05.

PFPN represents partial factor productivity of nitrogen, and AEN represents agronomic efficiency of nitrogen.

### 3.6 Regression analysis and structural equation model

**Figure 7:**
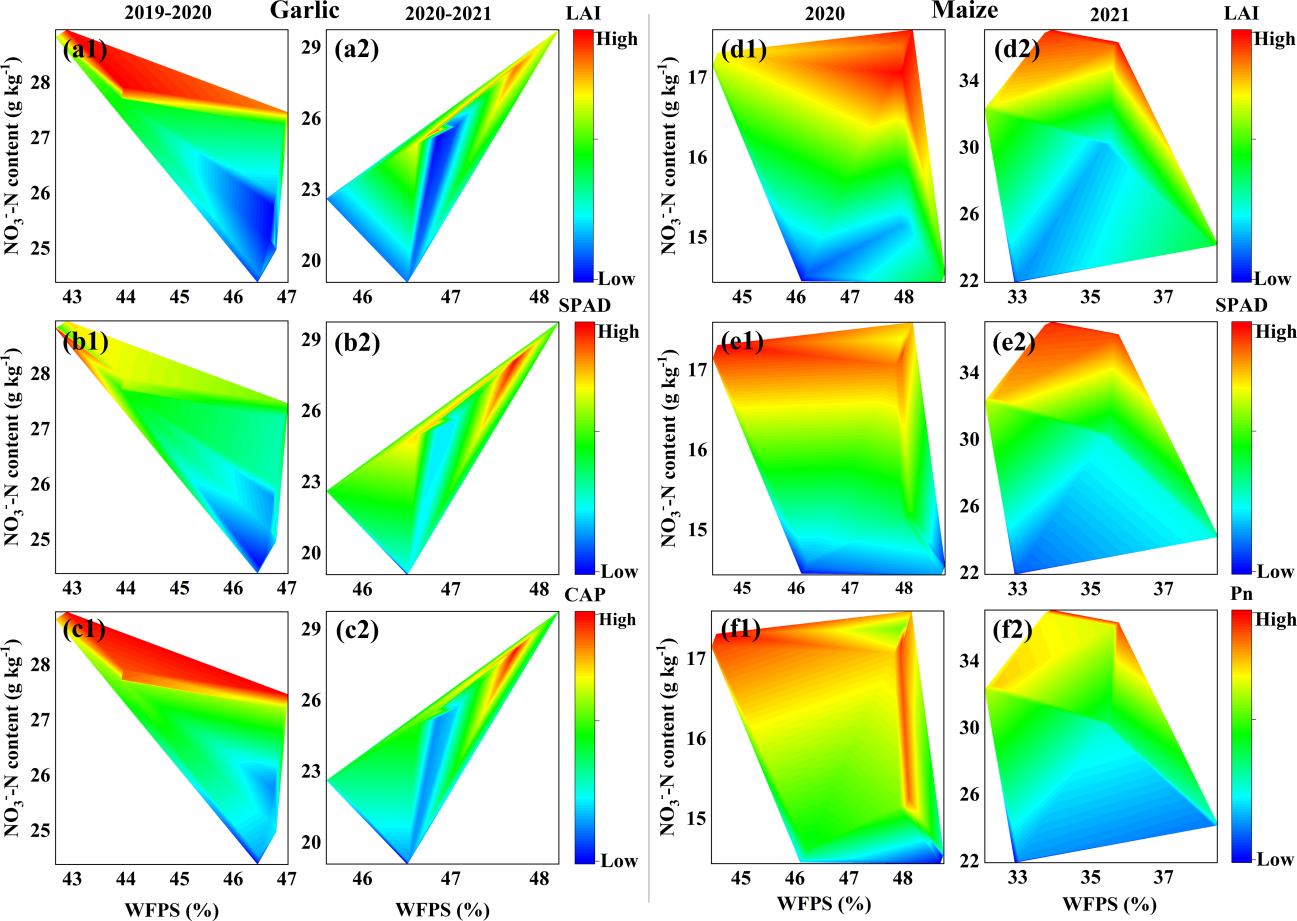
Effects of soil WFPS and NO_3_^−^–N concentration on leaf area index, SPAD and net photosynthetic rate.

Regression analysis indicated that the effects of soil WFPS and NO_3_^−^–N on leaf LAI, SPAD, and CAP (Pn) were similar, but there were differences in the effects on the two crops. The photosynthetic characteristics of maize showed similar responses over 2 years, with higher NO_3_^−^–N being a more important factor in improving plant photosynthetic characteristics compared to WFPS (**Figures 7d1–f2**). The difference in N application methods between the 2 years results in different responses of photosynthetic characteristics of garlic leaves to WFPS and NO_3_^−^–N. In 2019–2020, NO_3_^−^–N was the main influencing factor, while in 2020–2021, the photosynthetic capacity of plants was jointly affected by WFPS and NO_3_^−^–N (**Figures 7a1–c2**).

**Figure 8:**
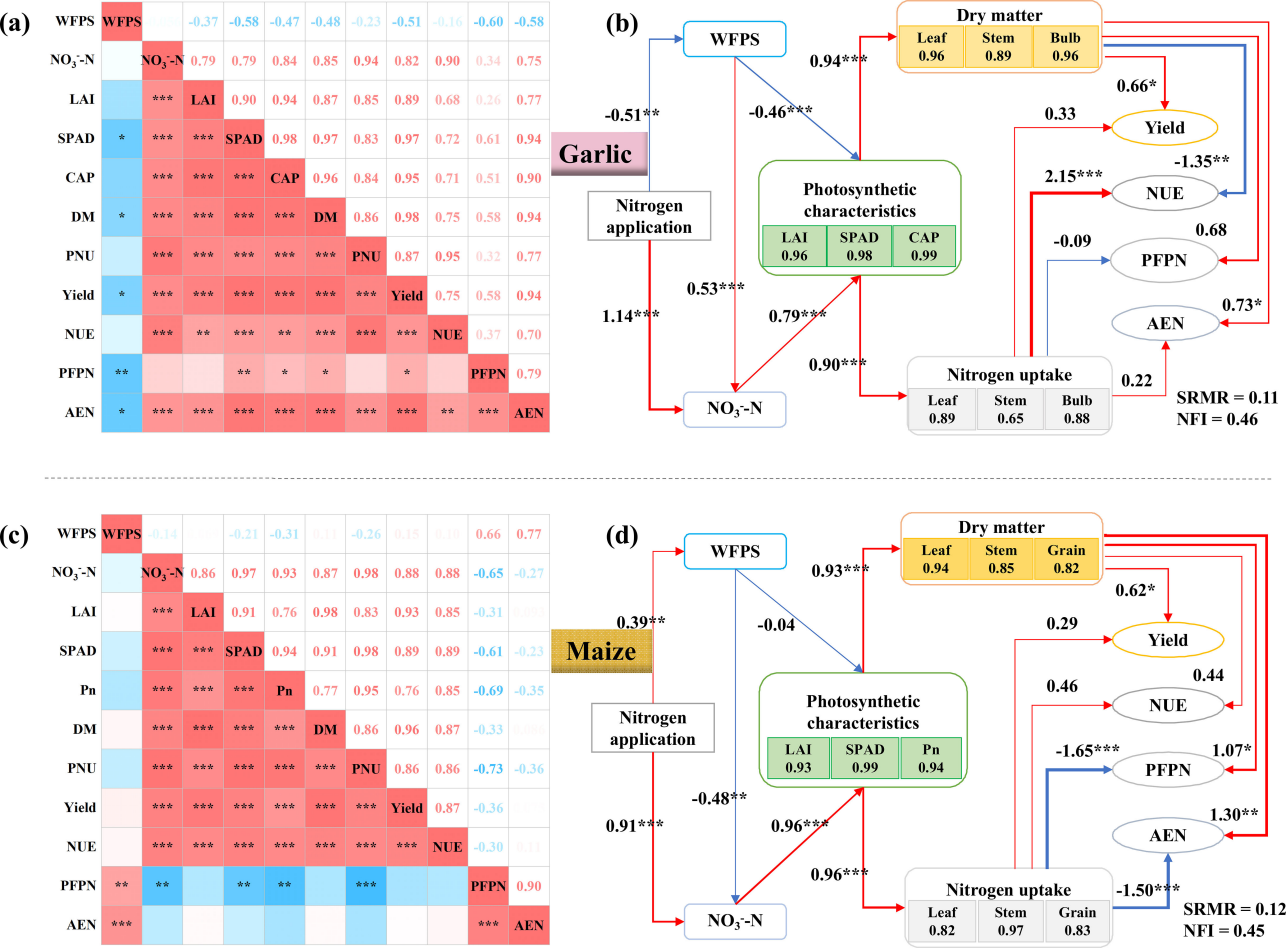
The effects of N application on soil WFPS and NO_3_^−^–N, leaf photosynthetic characteristics, dry matter and nitrogen distribution, and crop yield were analyzed by correlation analysis and structural equation. *, **, and *** represent significant correlation at 0.05,0.01,0.001 levels, respectively. The red arrows in the Fig. b and d indicate positive effect, and the blue arrows indicate negative effect. The thickness of the line indicates the level of influence. NUE represents nitrogen use efficiency, PFPN represents partial factor productivity from applied N, and AEN represents agronomic efficiency of applied N.

Path analysis showed that during the growth season of garlic and maize, increasing N application increased the NO_3_^−^–N, enhanced the photosynthetic capacity, and thus increased the dry matter and N absorption of the plants. Increasing N application decreased the soil WFPS in the garlic growing season, but enhanced plant photosynthesis (**Figure 8a**). Increasing N application increased the soil WFPS in the maize growing season and decreased the NO_3_^−^–N, but the increased WFPS had no significant effect on the plant’s photosynthetic capacity (**Figure 8b**). Compared to N uptake, the increase in crop dry matter quality was the main factor in improving the yield of the two crops, and the effects of dry matter quality and N uptake on the NUE of the two crops were different. During the garlic growing season, the increase in N uptake by plants increased NUE, while the increase in dry matter mass decreased NUE. During the growing season of maize, the increase in N uptake and dry matter mass of plants promoted NUE. The increase in dry matter quality increased the PFPN and AEN of garlic and maize, while the increase in N uptake decreased the PFPN and AEN of maize, but the impact on garlic was relatively small. In summary, path analysis revealed that N application influenced crop growth mainly through changes in NO_3_^−^–N and photosynthetic traits, with contrasting pathways between garlic and maize seasons.

## 4 Discussion

### 4.1 Nitrogen application rate affects the spatiotemporal distribution of soil NO_3_^−^–N

This study demonstrated that N reduction significantly decreased NO_3_^−^–N concentration in the 0–40-cm soil layer, with its dynamics exhibiting distinct seasonal and interannual variations. Controlling N application can regulate the content and distribution of NO_3_^−^–N in soil from the source (Guo et al., 2023; Liu et al., 2024). In this study, N reduction significantly decreased topsoil (0–40 cm) NO_3_^−^–N concentrations (**Figure 4**). However, seasonal and interannual variations in NO_3_^−^–N persisted due to crop functional traits and climatic conditions. WFPS emerged as the primary environmental regulator governing NO_3_^−^–N dynamics. Although elevated soil moisture typically enhances nitrification, it concurrently amplifies NO_3_^−^–N leaching potential (Liang et al., 2022; Krevh et al., 2023). During maize cultivation periods with high WFPS, 0–40-cm soil NO_3_^−^–N levels decreased significantly compared to low-moisture phases, indicating moisture-driven leaching as a key depletion mechanism (Zhang et al., 2021; Zuo et al., 2023). Affected by crop attributes, although the amount of N applied in the garlic growing season was higher than that in the maize growing season, the NO_3_^−^–N in garlic soil continued to remain at a low level. In previous studies, we found that the inhibition of garlic on soil urease activity reduces the substrate concentration of nitrification, thereby reducing the NO_3_^−^–N (Liu et al., 2023). Therefore, due to significant differences in soil nutrients, moisture, and growth characteristics among crop types in multiple cropping systems, it is necessary to analyze the growth changes of each crop during the season separately.

### 4.2 Nitrogen application rate changes the photosynthetic capacity of crops

The N application significantly enhanced the photosynthetic performance (LAI, SPAD, and Pn) of both garlic and maize by increasing soil NO_3_^−^–N availability. A significant negative correlation was observed between WFPS and garlic photosynthetic capacity, whereas no such correlation was found for maize. N enhances leaf photosynthetic capacity, promoting photosynthetic product synthesis and accumulation while increasing leaf area to improve light energy interception (Wang et al., 2023a; Qiang et al., 2025). This study revealed strong correlations between LAI, SPAD values, and net photosynthetic rate (Pn) in both garlic and maize growing seasons. Structural equation modeling confirmed that N application significantly improves crop photosynthetic performance through increased soil nitrate (NO_3_^−^–N) availability. These findings indicate that appropriate N optimization can enhance photosynthetic capacity in garlic–maize systems. Notably, WFPS showed a significant negative correlation with garlic photosynthetic capacity, but no correlation with maize (**Figure 8**). We propose that high WFPS triggers NO_3_^−^–N leaching from the 0–40-cm layer, disproportionately affecting shallow-rooted garlic, whereas deep-rooted maize compensates through subsoil nutrient acquisition (Jia et al., 2020; Zhang et al., 2022; Te et al., 2023). While previous studies have indicated that synergistic water–nitrogen coupling enhances crop photosynthesis (Hong et al., 2022), our results demonstrate that NO_3_^−^–N availability exerts greater influence than WFPS in garlic–maize systems. Both crops achieved photosynthetic maxima (LAI, SPAD, and Pn) under elevated NO_3_^−^–N regimes. Garlic exhibited pronounced annual variation in photosynthetic sensitivity to WFPS/NO_3_^−^–N interactions between seasons. Maize, conversely, approached photosynthetic maxima when NO_3_^−^–N was sufficient, irrespective of WFPS fluctuations. We propose that this divergence stems from differential root architectures: garlic’s shallow root system creates surface soil dependency, whereas maize supplements nutrient uptake through subsurface water acquisition from deeper horizons (Chen et al., 2023; Wu et al., 2022). The above analysis also indicated that there may be differences in the root function of maize distributed in the surface and deep layers of soil, which was consistent with the study by Zhang et al. (2021). Future studies employing *in situ* root imaging techniques could further validate this inference. Optimizing N availability is paramount for both crops, but ensuring good drainage or managing irrigation may be particularly critical for maximizing garlic photosynthesis and N uptake in seasons with high precipitation risk.

### 4.3 Nitrogen application rates shape the redistribution of dry matter and nitrogen uptake by crops

Strategic N reduction optimized the allocation of dry matter and N to the grains of maize. However, severe N limitation promoted dry matter and N retention in vegetative tissues (stems), limiting grain accumulation. In garlic, N reduction increased the stem dry matter to N uptake ratio and foliar N concentration. Strategic nitrogen (N) reduction curtails excessive vegetative growth, enhancing the allocation of photosynthetic assimilates and nitrogen to grains, thereby increasing crop yield (Yang et al., 2020; Liu et al., 2023). This study demonstrates that moderate N reduction during maize cultivation optimizes grain dry matter-to-N uptake ratios. However, severe N limitation (G_240_M_130_) conversely promoted dry matter accumulation in vegetative tissues while elevating stem N concentration, significantly limiting grain dry matter accumulation. This reveals a clear nitrogen threshold effect: moderate reduction (from M_240_ to M_175_) benefits grain allocation, whereas severe reduction (M_130_) crosses a threshold that triggers compensatory vegetative retention. We further observed that antecedent garlic-season N reduction enhanced stem-to-grain N transport during high-N maize phases. These findings establish that N reduction preferentially directs N remobilization from stems to grains, although extreme N deficiency triggers compensatory stem N retention to sustain maize growth (Qi and Pan, 2022; Lu et al., 2023). N absorption and allocation patterns differ fundamentally between garlic and maize. Reducing N application markedly elevated the stem dry matter-to-N uptake ratio in garlic while increasing foliar N enrichment. Regression analysis demonstrated organ-specific responses in dry matter and N absorption to N concentrations: garlic bulbs showed significant increases in dry matter and N accumulation with rising application rates, whereas maize exhibited grain yield suppression at elevated N levels but greater vegetative accumulation in stems and leaves (**Supplementary Figures 1a–b2**; see **Supplementary Figure 1**). Consequently, N management must be tailored to the species-specific sink strength and N demand of the target organ. A uniform N strategy is inefficient, as the optimal rate for maximizing bulb growth in garlic differs from that for optimizing grain yield and harvest index in maize.

### 4.4 Nitrogen application rate stabilizes crop yield and improves nitrogen utilization efficiency

Garlic and maize yields increased with elevated nitrogen (N) application rates (**Supplementary Figure 1c1, c2**). However, maize’s higher N demand threshold resulted in diminishing yield returns under high-N regimes compared to garlic (Liu et al., 2023). This underscores the practical significance of the identified N thresholds: surpassing the optimal range for maize leads to inefficient N use without proportional yield gain, defining a clear management ceiling. NUE responses to reduced N application diverged between crops: while strategic N reduction typically enhances crop NUE (Gu et al., 2021; Shen et al., 2023), our study found a 45 kg N ha^−1^ reduction during maize cultivation, which consistently improved maize NUE. Garlic showed interannual inconsistency due to inadequate N availability during high-yield seasons (**Supplementary Figure 1d1, d2**). Critically, maize exhibited improved PFPN through N reduction as yield increments plateaued under high N, whereas garlic maintained positive PFPN responses to added N during high-yield years. These divergent N efficiency metrics are directly linked to the physiological and allocation mechanisms discussed earlier. The improvement in maize NUE and PFPN under moderate N reduction aligns with the optimized dry matter and N partitioning to grains. Conversely, garlic’s variable NUE and sustained PFPN response reflect its distinct biomass partitioning strategy and higher sensitivity to soil N availability. Optimal N supplementation enhances vegetative growth and soil N acquisition (Li et al., 2023; Wang et al., 2023b). Maize AEN followed a parabolic trend—peaking at moderate N rates that stimulated soil N uptake but declining under excess application due to fertilizer dependency. Conversely, incremental N augmentation during garlic seasons improved soil N utilization.

Pathway analysis identified N application as the principal mechanism enhancing photosynthesis, dry matter synthesis, N uptake, and yield via increased 0–40-cm soil NO_3_^−^–N. However, the effect of WFPS on NO_3_^−^–N availability was species−specific, likely due to differences in plant growth patterns. Garlic biomass partitioning primarily governed NUE fluctuations, while maize efficiencies centered on PFPN and AEN, revealing distinct N utilization strategies rooted in resource allocation differences. Therefore, implementing crop-specific N management rather than a blanket application rate is crucial for optimizing yield and NUE in rotations involving crops with different N demands, sink types, and root architectures. This approach ensures that N availability is matched to the specific physiological and agronomic requirements of each crop in the system.

## 5 Conclusion

In the garlic–maize rotation, adjusting nitrogen (N) inputs significantly altered the soil NO_3_^−^–N levels within the 0–40-cm depth and its interaction with soil moisture (WFPS), thereby influencing photosynthetic capacity, dry matter formation, organ-level N partitioning, yield, and N-use metrics. Distinct crop-specific trade-offs were observed: garlic was more sensitive to N reduction, exhibiting decreased bulb allocation and yield under more severe N cuts. In contrast, a moderate reduction in maize (45 kg N ha^−1^ under the tested conditions) improved NUE with minimal yield penalty. Within the tested range, maize exhibited an apparent optimum at M_175_ (a reduction of 45 kg N ha^−1^), which enhanced efficiency and maintained yield, while further reduction to M130 (a reduction of 90 kg N ha^−1^) marked a risk threshold that impaired photosynthesis and grain dry matter/N allocation. Garlic had a lower tolerance threshold, with G_240_ (a reduction of 60 kg N ha^−1^) already reducing LAI, SPAD readings, CAP, and bulb allocation. Notably, reducing N in the preceding crop could diminish the yield of the subsequent crop, emphasizing the need for annual, system-level N budgeting rather than single-season decisions. Overall, these findings indicated that rotation-specific nutrient budgeting and diagnosis-based fertilization strategies (e.g., soil nitrate-informed and stage-specific adjustments) can sustain system productivity while enhancing NUE and lowering the risk of nitrate accumulation and its off-site impacts. This provided practical guidance for the environmentally responsible intensification of economic-grain multiple cropping systems.

## Data Availability

The original contributions presented in the study are included in the article/**Supplementary Material**. Further inquiries can be directed to the corresponding authors.
