# Supplementary material for: Optimizing nitrogen management in grain rotations: balancing retention and photosynthesis
Source: Front Plant Sci. 2026 Apr 2;17:1761467. doi: 10.3389/fpls.2026.1761467 (PMC13083001; doi:10.3389/fpls.2026.1761467)
Supplement: Supplementary file 1 [file Supplementaryfile1.docx]

**Table S1** Specific fertilization management

| Years | Treatments | Nitrogen fertilizer (kg ha^–1^ N) | | | |
| --- | --- | --- | --- | --- | --- |
|  |  | Maize | Garlic | | Total N |
|  |  | Base | Base | Topdressing |  |
| 2019/06-2020/05 | G_300_M_220_ | 220 | 0 | 300 | 520 |
|  | G_300_M_175_ | 175 | 0 | 300 | 475 |
|  | G_300_M_130_ | 130 | 0 | 300 | 430 |
|  | G_240_M_220_ | 220 | 0 | 240 | 460 |
|  | G_240_M_175_ | 175 | 0 | 240 | 415 |
|  | G_240_M_130_ | 130 | 0 | 240 | 370 |
| 2020/06-2021/05 | G_300_M_220_ | 220 | 150 | 150 | 520 |
|  | G_300_M_175_ | 175 | 150 | 150 | 475 |
|  | G_300_M_130_ | 130 | 150 | 150 | 430 |
|  | G_240_M_220_ | 220 | 120 | 120 | 460 |
|  | G_240_M_175_ | 175 | 120 | 120 | 415 |
|  | G_240_M_130_ | 130 | 120 | 120 | 370 |
| 2021/06-2022-05 | G_300_M_220_ | 220 | 150 | 150 | 520 |
|  | G_300_M_175_ | 175 | 150 | 150 | 475 |
|  | G_300_M_130_ | 130 | 150 | 150 | 430 |
|  | G_240_M_220_ | 220 | 120 | 120 | 460 |
|  | G_240_M_175_ | 175 | 120 | 120 | 415 |
|  | G_240_M_130_ | 130 | 120 | 120 | 370 |

G represents garlic and M represents maize. G_300_M_200_, G_300_M_175_, G_300_M_130_, G_240_M_220_, G_240_M_175_, and G_240_M_130_ represent the N input treatment combinations in the garlic and maize seasons.

Table S2 Crop yield and aboveground nitrogen uptake under nitrogen-free treatment

| Treatments | Crops | Yield  (kg ha^-1^) | Nitrogen uptake  (kg ha^-1^) |
| --- | --- | --- | --- |
| G_0_M_0_ | 2019-2020 Garlic | 2840.0 | 36.7 |
|  | 2020 Maize | 3310.4 | 112.6 |
|  | 2020-2021 Garlic | 10873.3 | 45.4 |
|  | 2021 Maize | 3982.0 | 99.2 |

G represents garlic and M represents maize.


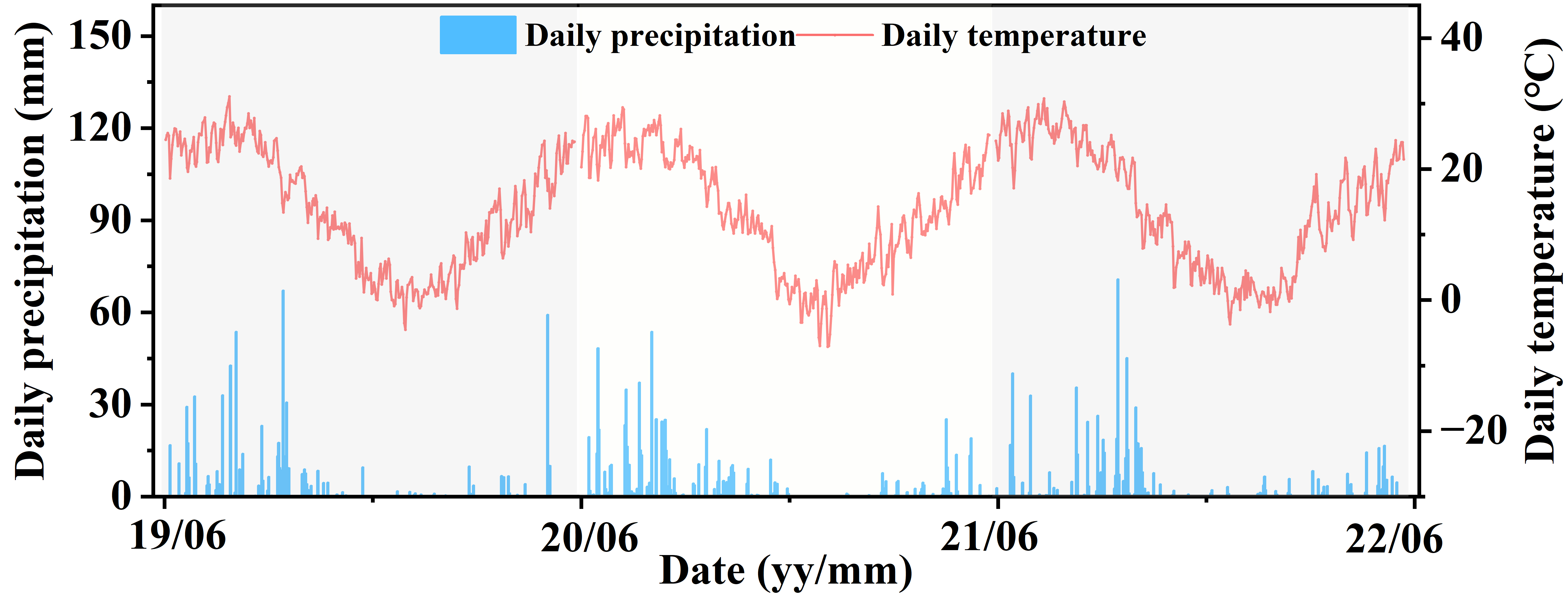


Figure S1 Precipitation and daily average temperature during the experiment


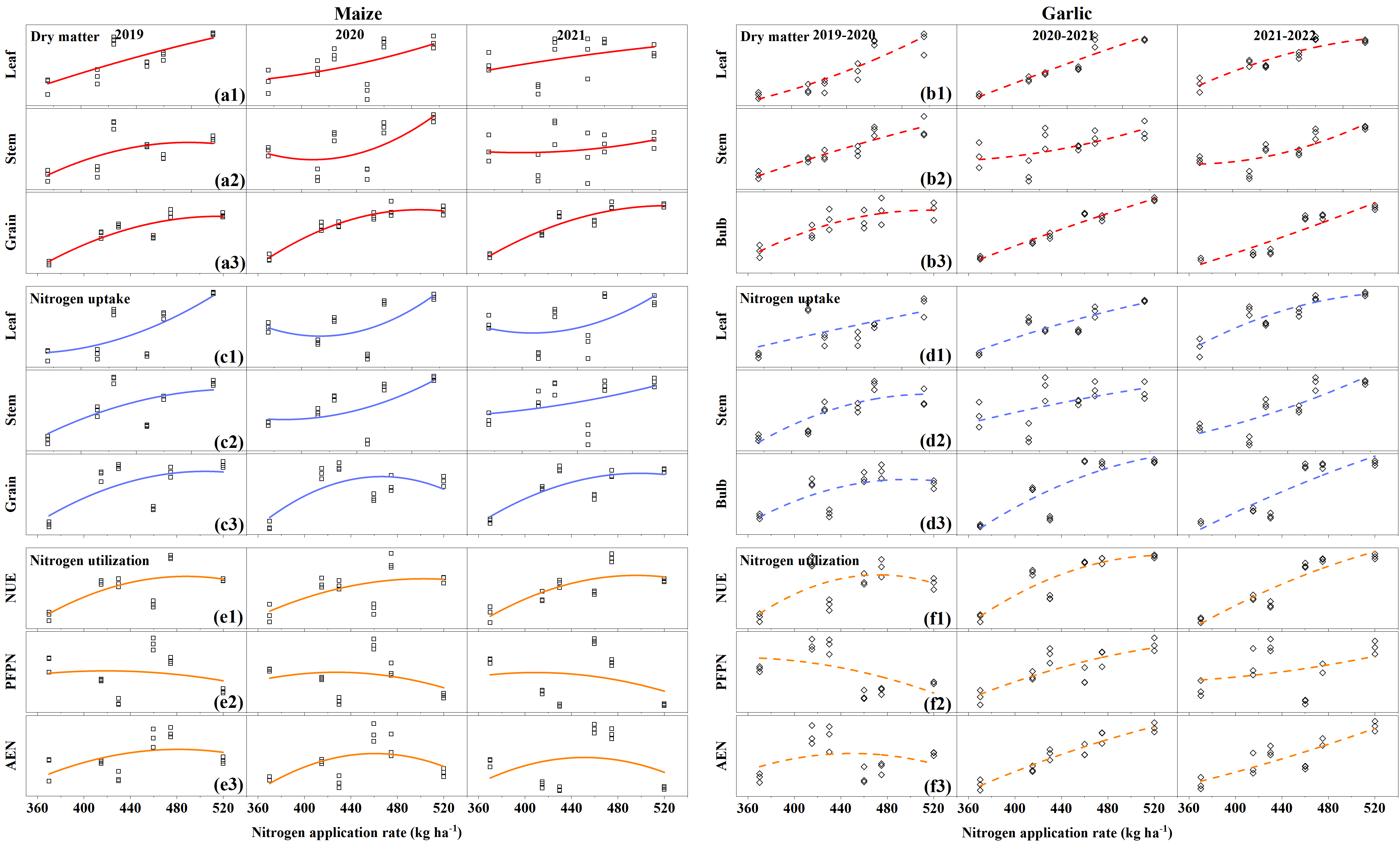


Figure S2 Regression analysis of crop index and nitrogen concentration. NUE represents nitrogen use efficiency, PFPN represents partial factor productivity of nitrogen, and AEN represents agronomic efficiency of nitrogen.


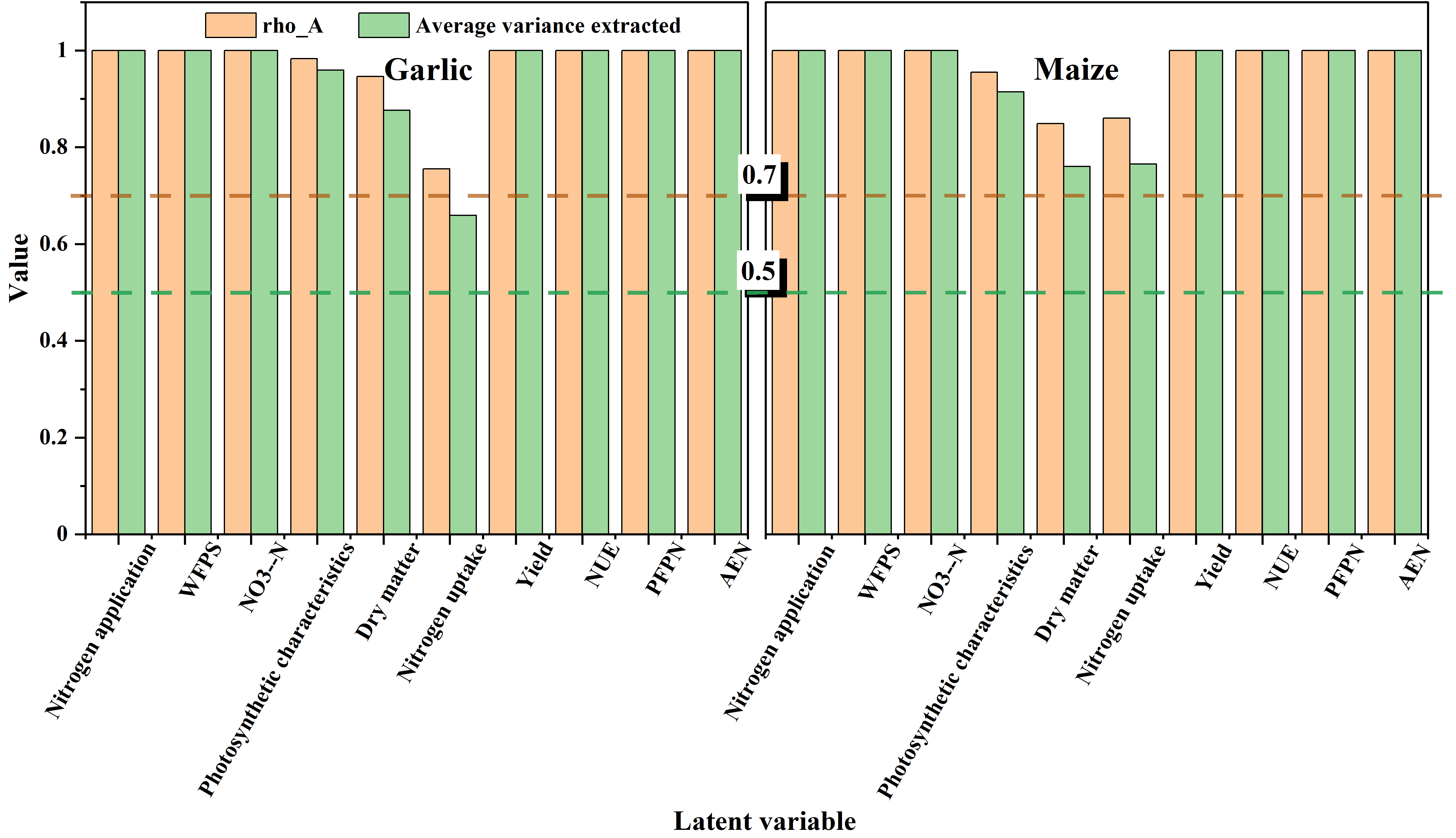


Figure S3 Test the reliability and validity of latent variables in structural equation model.

The yellow and green lines in the figure are the lowest values of reliability and validity test respectively. Each latent variable exceeds the minimum value, which meets the requirements of model construction.
